# Setting International Standards for the Management of Public Health Pesticides

**DOI:** 10.1371/journal.pmed.1001824

**Published:** 2015-05-12

**Authors:** Henk van den Berg, Rajpal Singh Yadav, Morteza Zaim

**Affiliations:** 1 Laboratory of Entomology, Wageningen University, Wageningen, the Netherlands; 2 Vector Ecology and Management, Department of Control of Neglected Tropical Diseases, World Health Organization, Geneva, Switzerland

## Abstract

Rajpal Singh Yadav and colleagues describe current and future challenges for the management of pesticides used in public health.

**Summary Points**

- Recent developments have highlighted the urgency of sound management of public health pesticides in vector-borne–disease–endemic countries.
- Major shortcomings are evident in national-level management practices throughout the pesticide life cycle from production to disposal; these shortcomings will adversely affect the cost-effectiveness and increase the risks of pesticides used.
- A major thrust has occurred towards developing international standards for improvement of public health pesticide management and towards expanding WHO’s global network on pesticide evaluation.
- However, to face current and future challenges, such as insecticide resistance in malaria vectors, the global capacity for evaluation of new insecticide products and vector-control tools should be further enhanced.
- Another area requiring urgent attention is the actual adoption and implementation of the recommended standards, calling for support to strengthen policy, legislation, and capacity.

## Introduction

Pesticide use has been the mainstay control of disease vectors and pests of importance to public health [1–3]. However, three recent developments have raised concern over how pesticides used for public health (which include vector-control pesticides, household insecticides, and professional pest-management pesticides [4]), are being regulated, managed, and monitored in affected countries.

Disturbing reports on the urban trade in substandard, poorly labelled, and highly toxic pesticide products, coined “street pesticides,” are probably the tip of the iceberg of unregulated products that are currently available to the public in developing countries for household use, as the problem is likely to be widespread [5,6].

Recent increases in vector-control insecticide use, particularly in malaria control programmes, have in many countries induced high levels of insecticide resistance, particularly to pyrethroids, which could seriously undermine the efficacy of vector-control interventions [7–10]. Insecticide resistance can partly be attributed to unsound use in public health and agriculture. The global use of vector-control insecticides is being dominated by pyrethroids [10,11], which is a major concern because pyrethroids are currently the only approved pesticides for manufacturing long-lasting insecticidal nets. Decisions made on the selection and use of insecticides or alternative methods, as well as coordination between sectors, have a direct bearing on the effectiveness, efficiency, and sustainability of vector control [12,13].

Moreover, country-level inventories of pesticide stocks have shown the existence of large stockpiles of obsolete vector-control insecticides and empty pesticide containers, often at highly contaminated sites, posing risks to human health and the environment [14]. Clearly, poor management practices have contributed to the accumulation of obsolete stocks.

These developments highlight the urgency of sound management of public health pesticides. A globally accepted norm for pesticide management has been established in the International Code of Conduct on Pesticide Management, which has recently been updated to strengthen health and environmental protection [15]. To assist countries in implementation of the Code of Conduct, WHO, in 2003, drafted a framework for public health pesticide management [4] and, since 2007, maintained a joint programme on pesticide management with the Food and Agriculture Organization of the United Nations (FAO) in support of member states. Additionally, several international legal instruments are in place to assist countries in controlling the use, trans-boundary movements, international trade, and disposal of hazardous pesticides [16–18].

In this paper, we discuss the development of international standards and guidelines on best practices as one of the strategies for strengthening pesticide management in public health. We have previously discussed strategies for pesticide management at the regional and country levels [19].

## Key Issues in Pesticide Management

The life-cycle concept of pesticide management refers to the legislation, regulatory control, and management practices necessary for the stages of a pesticide, from production to disposal or degradation [15,20]. Without comprehensive and sound management practices, the effectiveness of pesticides will be compromised, risks elevated, and resources wasted.

Foremost, national registration schemes, supported by legislation, are required that exercise control over public health pesticide products, their acceptable uses, and quality standards. A global survey indicated, however, that many countries lacked essential procedures for registration of public health pesticides; for example, 40% of countries lacked guidelines on the registration process [21].

Moreover, pesticide procurement practices should be supported by guidelines, legal provisions, and controls to obtain high-quality products in a transparent manner. However, survey data showed that guidelines on procurement were lacking in half of the countries [22].

Pesticide transport and storage should ensure safety and prevent spillage and accumulation of obsolete stocks; pesticide distribution and trade should be licenced; and pesticide disposal should comply with existing standards. Nevertheless, legislation on key aspects of pesticide management, such as transport, labelling, storage and disposal, was lacking in many countries; the situation in Africa, where vector-control insecticide use showed a significant recent increase, was below the average, with half of the countries lacking legislation on safe transport or disposal. Also, the enforcement of pesticide regulations was inadequate in half of the countries [21].

The application of pesticides must be supported by training on best practices, evidence-based decision-making, personal protection measures, and record keeping. Yet, the global survey showed that training was lacking among part of staff responsible for decision-making and implementation of vector control, especially in Africa [22].

Routine monitoring of pesticide exposure, poisoning cases, and insecticide susceptibility is essential; however, applicator exposure was monitored in only 26% of countries [22]. Pesticide quality control was equally poor, with inadequate laboratory capacity and substandard and/or counterfeit public health pesticides being a concern in most countries.

Furthermore, information exchange and intersectoral coordination are vital to ensure consistency and efficiency in pesticide management, for instance, by coordinating the management of pesticide resistance between sectors of agriculture and health. However, a separate study reported poor intersectoral coordination on pesticides [19].

In short, the global situation on public health pesticide management showed critical shortcomings across the entire spectrum of practices.

## Authoritative Guidelines

Recommended practices in public health pesticide management should be available to countries in the form of international guidelines that outline procedures, criteria, and requirements. In recent years, guidelines have been developed under the auspices of WHO pertaining to various components of pesticide management (Table 1). Nonetheless, guidelines are missing for practices such as storage, transport, and distribution; pesticide handling; larviciding; and public education, whilst several existing guidelines may be outdated. Future efforts should address these gaps.

**Table 1:** WHO and/or FAO global authoritative guidelines in relation to the individual components of public health pesticide management [23].

Component	Sub-component	Guidelines published
Registration	Registration of pesticides	2010
	Data requirements for pesticide registration	2013
Procurement	Procuring public health pesticides	2012
	Pesticide advertising	2010
Management	Formulation and repackaging	N/a
	Storage and transport	N/a
	Distribution	N/a
	Licensing	N/a
Application	Indoor Residual Spraying	2013
	Equipment for vector control	2010
	Space spray application	2003
	Personal protection and pesticide handling	N/a
	Larviciding	N/a
	Decision-making for judicious use of insecticides	2005
Disposal	Prevention of accumulation of obsolete pesticides	1995
	Management of empty pesticide containers	2008
Monitoring	Post-registration pesticide monitoring	N/a
	Reporting incidents of pesticide exposure	2009
	Test procedures for insecticide resistance monitoring	2013
Quality control	Quality control of pesticides	2011
	Quality assurance of national laboratories	2005
Communication	Public education	N/a
	Information exchange	N/a

N/a indicates that guidelines are not yet available.

To enable national regulatory authorities to register candidate pesticide products, specialized laboratories should produce reliable and representative product data regarding use efficacy and human and environmental risks. For this purpose, internationally agreed-upon procedures on efficacy testing and risk assessment should be available for each pesticide application method used to control vectors and pests of public health importance.

Major recent advances have been made in guidelines development on efficacy testing and risk assessment aiming to harmonize procedures to generate data needed for registration and use of products in disease-endemic countries (Table 2). For the main vector-control application methods, these guidelines are available. Gaps remain in globally harmonized risk assessment for repellents, household insecticides, and specific groups of public health pests. Another gap is the efficacy testing against vectors that have already developed resistance. Moreover, novel application methods, such as durable wall lining, spatial repellents, and insecticide-treated clothing, will require guidelines once their role is established.

**Table 2:** Global authoritative guidelines on efficacy testing and risk assessment in relation to each vector-control application method or tool [23].

	Risk assessment	Efficacy testing	
Application method/tool	Guidelines published	Guidelines published	Target
Long-lasting insecticidal nets	2012	2011	Malaria vectors
		2013	Malaria vectors
Indoor residual spraying	2011	2001	Vectors of Chagas disease
		2006	Malaria and leishmaniasis vectors
Larviciding	2011	2005	Mosquitoes
Space spraying	2011	2001	Dengue vectors
		2009	Vectors and pests of public health importance
Aircraft disinsection	2014	2012	Disease vectors
Spatial repellents	N/a	2013	Mosquitoes
Repellents for human skin	N/a	2009	Mosquitoes
Household insecticides	N/a	2009	Mosquitoes
Pest control products (e.g., houseflies, rodents, bedbugs, snails)	N/a	N/a	−
New tools (e.g., durable wall lining; insecticide-treated clothing)	N/a	N/a	−

N/a indicates that guidelines are not yet available.

## Pesticide Evaluation and Specification

Pesticides used in public health should be suited for their intended purposes and be of high quality. Substandard, counterfeit, and adulterated pesticides [24] will not have the intended efficacy, thus wasting resources while posing unforeseen risks to human health and the environment [5]. Hence, international pesticide quality standards should be available to disease-endemic countries so that these standards can be incorporated in national-level regulatory control measures. Monitoring and enforcement of pesticide quality standards is the responsibility of each country.

WHO’s Pesticide Evaluation Scheme (WHOPES) oversees the phased evaluation of pesticide products and produces international recommendations to support national regulatory authorities and disease control programmes in product registration and use [25]. Manufacturers voluntarily submit their products for evaluation and bear the costs incurred. The evaluation comprises laboratory testing on inherent properties and cross-resistance properties of the chemical (Phase I), small-scale field studies on efficacy (Phase II), and large-scale field studies on efficacy and operational acceptability (Phase III).

Acceptable products are submitted for development of pesticide specifications, which describe physical and chemical characteristics to provide countries with the point of reference for quality control [26]. The specification of pesticide products is developed jointly by WHO and FAO.

**Fig 1:**
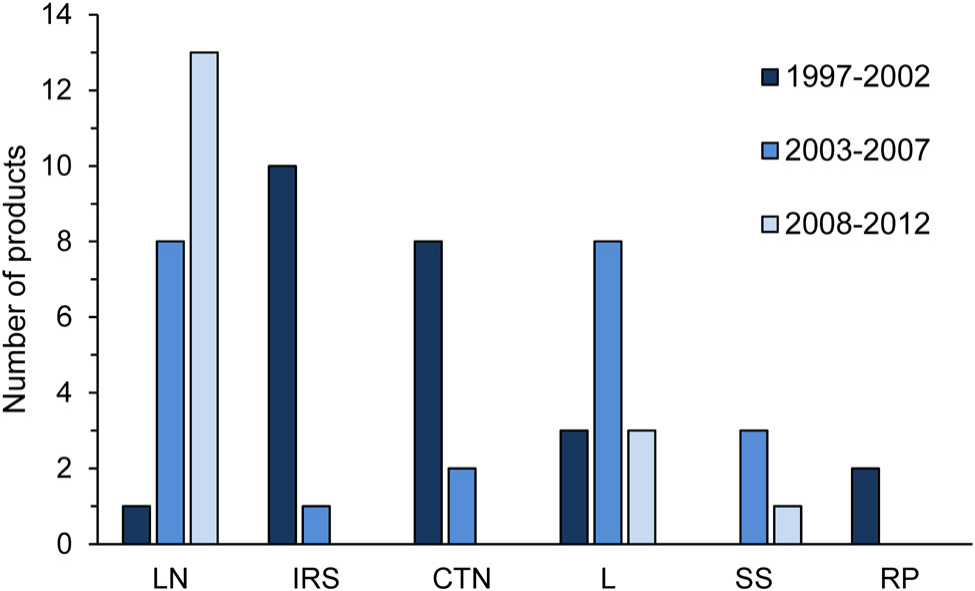
Number of products per application method that have been evaluated by the WHOPES Working Group in its annual meetings, pooled over three periods [23]. LN = long-lasting insecticidal nets; IRS = indoor residual spraying; CTN = conventionally treated nets; L = larviciding; SS = space spraying; RP = repellent.

On average, the scheme completes the evaluation of four new products per year. Over the past fifteen years, there has been a shift in submission of products by industry, from indoor residual spraying and conventionally treated nets towards long-lasting insecticidal nets (Fig 1).

WHOPES operates through a global network of WHO Collaborating Centres and other partner institutions, each with its designated task. The network has recently expanded, from eight institutions in 2009 to nineteen in 2013 [27]. New tasks, such as the production of insecticide susceptibility kits and laboratory testing of pesticides, have been added to the network, and technical capacity has been enhanced. Also, stricter requirements have resulted in a substantial intensification of the evaluation process; in 2012, the number of field sites required for Phase II and III studies was increased from two sites previously to three sites per evaluated product, with exceptions for certain products. The most commonly designated task is field studies on vector-control products (Table 3).

**Table 3:** Designated tasks in the WHOPES global network of institutions [27].

Designated task of institution	Institution
Quality control of pesticides	Walloon Agricultural Research Centre, Gembloux, Belgium*
Testing of insecticide application equipment	International Pesticide Application Research Centre, Ascot, UK*
Laboratory testing and evaluation of pesticides	National Institute of Malaria Research, Delhi, India*
	Laboratoire de Lutte Contre les Insectes Nuisibles, Montpellier, France*
Vector surveillance and management	Department of Vector Biology and Control, Changping, China*
Research and training in lymphatic filariasis and integrated methods of vector control	Vector Control Research Centre, Pondicherry, India*
Production of laboratory tools for testing insecticide susceptibility	Vector Control Research Unit, Penang, Malaysia
Field studies on vector control products	Centers for Disease Control and Prevention, Atlanta, USA
	Centre for Disease Control, Beijing, China
	Instito National de Salud Publica, Tapachula, Mexico
	Institut de Recherche pour le Développement, Cotonou, Benin
	Institute of Tropical Medicine, Antwerp, Belgium
	Kenya Medical Research Institute, Nairobi, Kenya
	London School of Hygiene and Tropical Medicine, UK
	Manatee County Mosquito Control, Palmetto, Florida, USA
	Medical Research Council, Fajara Nr Banjul, the Gambia
	National Institute of Malaria Research, Delhi, India*
	Noguchi Memorial Institute for Medical Research, Accra, Ghana
	Regional Medical Research Centre, Dibrugarh, Assam, India
	University of the Witwatersrand, Johannesburg, South Africa

* WHO Collaborating Centre, designated through ministerial agreement and with fixed workplan. Other partner institutions are contracted on an ad-hoc basis. WHO applies the same quality control standards to all institutions.

## Way Forward

A major thrust towards developing international standards for public health pesticide management has occurred with recent donor support, primarily from Bill & Melinda Gates Foundation. The international standards are expected to assist national authorities and programme managers in pesticide management, but further work is needed to face future challenges and address remaining gaps.

Looking ahead, disease vectors and pests will continue to develop resistance to the pesticide products being used. There is a limited arsenal of pesticides available for public health [28]; most of these are molecules that were originally developed for agriculture. New methods, approaches, and strategies of control are also urgently needed, as illustrated by reports on increased relative importance of outdoor transmission of malaria following the scaling up of indoor residual spraying and long-lasting insecticidal net interventions [29].

To meet the challenge of insecticide resistance in disease vectors, the Innovative Vector Control Consortium (IVCC), a product-development partnership, is developing new insecticide products and tools [10]. In 2013, WHO launched the Vector Control Advisory Group (VCAG) on new tools and paradigms, to foster the development and adoption of new methods and strategies of vector control. These and related initiatives are expected to produce a new wave of submissions of products and methods for evaluation.

An increase in product submissions, the stepped-up process of evaluation, and the demand to reduce the time of bringing new products to market should be matched by a continued strengthening of the WHO global network for pesticide evaluation and for development of specifications, guidelines, and standard operating procedures. Regarding the evaluation of novel application methods, institutions with the required expertise will need to be added to the global network.

International standards on pesticide management are of little relevance if not adopted and implemented by disease-endemic countries. There are strong indications that these countries are receptive to adopting standards on registration or pesticide quality as their national requirements [21,22]. However, considering the dire situation on pesticide management in public health, increased advocacy is urgently needed on the importance of risk reduction and safeguarding effectiveness of pesticides. To help resource-poor countries strengthen their policy, legislation, and capacity for sound pesticide management in public health, substantial external support will be necessary.

In a separate contribution, we evaluated three strategies for strengthening pesticide management at country level: regional policy development to raise awareness in countries, in-depth country support for situation analysis and action planning, and thematic support on a prioritized aspect of pesticide management across countries [19]. These three strategies appear to be largely synergistic, by initiating political support and addressing country-specific priority gaps while also tackling common problems across countries. FAO is actively supporting regional coordination and work-sharing for pesticide registration, covering agricultural and public health pesticides, and other aspects of pesticide management also need coordination at regional level [30].

At the global level, the set of internationally agreed-upon standards, guidelines, and pesticide specifications should be further developed to guide individual countries in improving their legislation, regulation, and practices of pesticide management.

## Abbreviations

FAO: Food and Agriculture Organization of the United Nations
IVCC: the Innovative Vector Control Consortium
VCAG: Vector Control Advisory Group
WHOPES: WHO Pesticide Evaluation Scheme
